# Searching for an optimal AUC estimation method: a never-ending task?

**DOI:** 10.1007/s10928-014-9392-y

**Published:** 2014-10-15

**Authors:** Wojciech Jawień

**Affiliations:** Faculty of Pharmacy, Jagiellonian University in Kraków, ul. Medyczna 9, 30-688 Kraków, Poland

**Keywords:** AUC, Optimal sampling theory, Limited sampling strategy, Quadrature, Estimation, Minimax, Bioequivalence

## Abstract

An effective method of construction of a linear estimator of AUC in the finite interval, optimal in the minimax sense, is developed and demonstrated for five PK models. The models may be given as an explicit *C*(*t*) relationship or defined by differential equations. For high variability and rich sampling the optimal method is only moderately advantageous over optimal trapezoid or standard numerical approaches (Gauss-Legendre or Clenshaw-Curtis quadratures). The difference between the optimal estimator and other methods becomes more pronounced with a decrease in sample size or decrease in the variability. The described estimation method may appear useful in development of limited-sampling strategies for AUC determination, as an alternative to the widely used regression-based approach. It is indicated that many alternative approaches are also possible.

**Electronic supplementary material:**

The online version of this article (doi:10.1007/s10928-014-9392-y) contains supplementary material, which is available to authorized users.

## Introduction

The estimation of integral of a function, or area under the curve (AUC), plays an important role in biomedicine including in pharmacokinetic (PK) or toxicokinetic studies that are designed to estimate the integral of concentration of the investigated compound in plasma or tissue taken over time in a given interval.

$$\begin{aligned} AUC = \int _{t_1} ^{t_2} C \left( t \right) dt \end{aligned}$$

Within the framework of linear compartmental models AUC established after an intravenous administration is used to calculate the drug clearance. Regardless of which one of the possible linear models is valid, the result is determined solely by the drug dose and AUC. This is one of the reasons for AUC to be a central concept of the so-called model-independent pharmacokinetics. Regulatory institutions use AUC as a measure of extent of absorption in order to assess a bioequivalence of different formulations of the same drug [1–3].

Many authors have addressed the problem of practical determination of AUC. A few papers contain reviews of numerous algorithms designed to estimate this parameter [4, 5]. Their authors do not pay any particular attention to the choice of sampling times, assuming they are given a priori, maybe following a certain traditional pattern. On the other hand, several authors have investigated the optimal designs which should yield the most accurate results using specific approaches.

The optimal sampling is especially important if the number of measured concentrations is limited due to ethical and economical reasons. Duffull et al. searched for the optimal design with limited sampling for the log-trapezoid rule applied to the two-exponential equation [6]. A vast number of authors (MEDLINE reports about 200 papers [7]) developed limited sampling strategies for estimating AUC either of specific drugs, for instance cyclosporine [8, 9] or midazolam [10], or in the general situation [11].

Katz and D’Argenio found optimal sampling times for estimating the integral of bi- and triexponential equations using the trapezoid rule [12]. In their original form these results are of limited usefulness, since the authors have assumed fixed values of parameters of those equations. In practice such parameters are more or less uncertain (if they were certain then the exact AUC would also be known and no estimation would be necessary). The present paper extends, in several directions, the ideas of that work. Namely, the aims of this work are threefold:

1. Find an optimal sample schedule design for trapezoid rule under parameter uncertainty.
2. Find an optimal quadrature within the class of linear combination (LC) quadrature approximations [13]. This is to be achieved by simultaneous adjustment of both sampling design and coefficients (weights) of quadrature.
3. Evaluate obtained quadratures for five common PK models by means of simulation.

In order to reach these aims it is required to:

1. set up transparent criteria of optimality;
2. state necessary assumptions to make the problem tractable;
3. express it as an optimization problem (in this case a minimax problem);
4. invent an approach to practically solve this optimization problem;
5. implement it (as a numerical analysis task);
6. plan and execute optimum searches and simulations;
7. evaluate the results.

These points would be reported in the following sections after introducing the necessary background.

## Method

### Background: theory of point estimation

While concepts to be introduced here are quite general elements of a statistical decision theory, the presentation will focus on their application to the theory of point estimation or, even more specifically, to the estimation of *AUC* in PK models. Let $\varvec{\theta }$ be a vector of standard (primary) PK parameters. True *AUC* may be expressed as a function of these parameters, $AUC(\varvec{\theta })$.

An estimator of an unknown quantity is a function of observations that in some way approximates that quantity. As any experiment suffers from various nuisance factors, the observations do not follow any deterministic model exactly. Thus the estimation can be imperfect. One may intuitively expect that certain estimators can perform better than others. However, if one would like to transform this intuition into a scientific method, a rigorous criterion is needed that would enable comparison of estimators.

Towards this end, a statistical theory of point estimation [14] introduces a loss function $L ( \hat{Q}, \varvec{\theta })$ to assess the precision of an estimator $\hat{Q}$. A loss function is always non-negative and it should yield 0 if an estimation is exact, i.e. if $\hat{Q} = AUC (\varvec{\theta }).$ A quadratic loss function

$$\begin{aligned} L ( \hat{Q}, \varvec{\theta })= \left[ \hat{Q} - AUC \left( \varvec{\theta }\right) \right] ^2 \end{aligned}$$

is a typical choice, and it is one of two that will be considered here. The intuition behind this concept is rather simple: a wrong estimation causes a loss. The worse the estimate is, the higher the loss will be. Or: the closer the estimator value to the true AUC is, the lower the loss will be.

Another important concept is that of risk function. It is defined as an expectation of a loss. The expectation is taken over a joint probability distribution of all $\varepsilon _i$.

$$\begin{aligned} R \left( \varvec{\theta }\right) = E \left[ L ( \hat{Q}, \varvec{\theta })\right] \end{aligned}$$

The expectation of a continuous random variable $X$ is defined as the first moment of its probability density function $\varphi (x)$:

$$\begin{aligned} E \left[ X \right] = \int x \varphi (x) dx \end{aligned}$$

While this definition might appear somewhat abstract, the expectation has quite simple interpretation, due to a fundamental law of statistics: The Law of Large Numbers. If one repeatedly observes a quantity that is random, then the average result should tend to the expectation of that quantity (for a rigorous formulation of that law refer to any textbook on statistics, e.g. [15]). Thus, if one were to repeat estimation with a given estimator, then the average loss should tend to that estimator’s risk.

Estimators may be compared on a basis of their risk. Unfortunately, there is no estimator which is better than any other estimator for any parameter vector $\varvec{\theta }$ [14]. Nonetheless, an optimal estimator in that sense (called a uniformly optimal estimator) could be found if some restrictions were applied to the class of considered estimators. Perhaps the most popular one is the case of unbiased estimators with quadratic loss function. They are called minimum variance unbiased estimators (MVUE).

There can be little benefit from MVUE in pharmacokinetics, however, as unbiased estimators do not exist for standard PK models with usual parameters (or, at least, they remain unknown).

Despite these problems, a good or even the best estimator, according to reasonable criteria, can be constructed in a somewhat different manner. The choice between two standard solutions depends on whether $\varvec{\theta }$ is treated as a random variable with known distribution or as an unknown parameter. In the first case an estimator that minimizes expectation of the risk (over $\varvec{\theta }$ distribution) is searched for. It corresponds to a Bayesian approach. In the second case a maximum possible risk is minimized. That is a minimax problem.

The former approach requires a knowledge of statistical distribution of PK parameters, while in the second method one needs to only know the range of those parameters. In what follows, the latter choice is analysed in detail.

### Approach of Katz and D’Argenio

The trapezoid rule may be expressed by the following equation

1 $$\begin{aligned} Q=\sum _{i=0} ^n w_i C(t_i), \end{aligned}$$

where

2 $$\begin{aligned} w_0=&\frac{t_1-t_0}{2}; \; w_i=\frac{t_{i+1}-t_{i-1}}{2}, i=1,\ldots ,n-1;\nonumber \\ w_n=&\frac{t_n-t_{n-1}}{2}. \end{aligned}$$

In practice, an integral is calculated based on measured concentrations. Assume the integrand follows a certain PK model with a parameter vector $\varvec{\theta }$. Thus, what is measured can be expressed by the equation:

$$\begin{aligned} \hat{C} _i=C\left( t_i,\varvec{\theta }\right) +\varepsilon _i \end{aligned}$$

where $\varepsilon _i$ is a random error. The result

$$\begin{aligned} \hat{Q} =\sum _{i=0} ^n w_i \hat{C}_i \end{aligned}$$

is therefore a random variable. It may be considered as a linear estimator of an unknown integral *AUC*.

In their paper Katz and D’Argenio proposed “selecting observation times so to minimize the expected value of the squared difference between the estimator and the exact value of the integral”. These authors assumed specific parameters of a multiexponential equation (i.e. they fixed $\varvec{\theta }$) and numerically found a minimum over a vector of sampling times, $\varvec{t}$:

3 $$\begin{aligned} \min _{\varvec{t}} E(\hat{Q} - AUC)^2. \end{aligned}$$

In terms of previous subsection a sampling schedule that minimizes the risk of trapezoid rule estimator was found. In order to include a variance model of $\varepsilon _i$ they used the decomposition of the expectation of the squared error into a variance of estimator and its squared bias

4 $$\begin{aligned} E \left[ \left( \hat{Q} - AUC \right) ^2 \right] = V \left( \hat{Q} \right) + \left[ AUC - E \left( \hat{Q}\right) \right] ^2, \end{aligned}$$

the well-known result (for derivation see, for instance, Lehmann [14] or Katz and D’Argenio [12]). If all $\varepsilon _i$ are independently distributed with the mean 0 and variance $\sigma _i ^2$, then

5 $$\begin{aligned} E\left( \hat{Q}\right) =\sum _{i=0} ^n {w_i C\left( t_i \right) }\end{aligned}$$

6 $$\begin{aligned} V\left( \hat{Q}\right) =\sum _{i=0} ^n {w_i ^2 \sigma _i ^2} \end{aligned}$$

Note that detailed knowledge of the statistical distribution of $\varepsilon _i$ is not required; any distribution with existing and known variance can be accepted. One way to make $\sigma _i^2$ known is to express it as a function of *C*. The heteroschedastic model with a constant coefficient of variation (*c*_*v*_) is often assumed in PK models. It will be followed in the present study.

7 $$\begin{aligned} \sigma _i = c_v C\left( t_i,\varvec{\theta }\right) \end{aligned}$$

As the specific values of parameters need to be assumed, AUC may be calculated based on them and there is no need to use concentrations at all. Katz and D’Argenio made a rudimentary analysis of how the precision of the trapezoid method in an optimal setting changes while changing some (not all) parameters. It may be done in a more systematic manner within the framework of minimax approach and this will be one extension the present paper makes to the ideas of Katz and D’Argenio.

### Optimal sample schedule design

Using the minimax approach the minimization of the risk for a given $\varvec{\theta }$ should be replaced by minimization of the maximum risk that can be obtained for any possible vector of parameters. Thus the problem in Eq. 3 should be rewritten as

$$\begin{aligned} \min _{\varvec{t}} \max _{\varvec{\theta }} R(\varvec{\theta }) = \min _{ \varvec{t}} \max _{\varvec{\theta }} E \left[ \left( \hat{Q} - AUC (\varvec{\theta }) \right) ^2 \right] . \end{aligned}$$

For highly variable drugs it might be more useful to minimize a relative rather than an absolute error. This corresponds to the division of the loss function *L* by the squared $AUC(\varvec{\theta })$.

$$\begin{aligned} L_r ( \hat{Q}, \varvec{\theta })= \frac{\left[ \hat{Q} - AUC \left( \varvec{\theta }\right) \right] ^2}{\left[ AUC \left( \varvec{\theta }\right) \right] ^2} \end{aligned}$$

An optimum based on the above loss function (let it be called relative, in contrast to an absolute function *L*) will be analysed in the present study. The corresponding risk function will be denoted by $R_r(\varvec{\theta })$ and an expression for the required optimum takes on the form:

$$\begin{aligned} \min _{\varvec{t}} \max _{\varvec{\theta }} R_r(\varvec{\theta }) = \min _{ \varvec{t}} \max _{\varvec{\theta }} E \left[ \left( \frac{\hat{Q} - AUC (\varvec{\theta })}{AUC\left( \varvec{\theta }\right) } \right) ^2 \right] . \end{aligned}$$

In bioequivalence studies *AUC* is being compared on a logarithmic scale; equivalently the comparison focuses on ratios and not differences of *AUC* values. Also, in clinical application, an estimation error of 10 units is certainly more important if the true *AUC* equals 50 units than in a case when it is as large as 300. In both situations use of a relative risk would be preferable over an absolute risk.

Substituting Eqs. 5 and 6 into Eq. 4 and taking into account Eq. 7 yields a useful expression for the risk function that is a subject of minimax optimization:

8 $$\begin{aligned} R_r ( \varvec{\theta }) =&\left\{ c_v^2 \sum _{i=0}^n w_i^2 [C(t_i,\varvec{\theta })]^2 + \right. \nonumber \\&\left. +\left[ AUC(\varvec{\theta })-\sum _{i=0}^n w_i C(t_i,\varvec{\theta })\right] ^2 \right\} \nonumber \\&/\;\left[ AUC (\varvec{\theta }) \right] ^2 \end{aligned}$$

This is a nested problem: there is a maximization over $\varvec{\theta }$ within a minimization over $\varvec{t}$. It means that for each trial sampling schedule the maximization in $\varvec{\theta }$ has to be conducted and finally that sampling schedule which yielded the smallest maximum is to be chosen. An optimization is constrained on both levels: PK parameter values should stay within a reasonable range; sampling times should be arranged in ascending order and they should be included in the integration interval. This constrained optimization problem cannot be solved analytically and an application of numerical algorithms is required. The optimization seems to be one of the most difficult branches of numerical analysis. There is always the possibility that the solution found would appear suboptimal. In order to minimize this possibility, the advanced methods are required using as much information as is available. This is especially important on an inner level: unstable results may mislead outer level optimization routine and thwart convergence.

The necessary information includes first and second derivatives of the inner objective in $\varvec{\theta }$, since they describe important geometrical properties of the hypersurface along which the maximum is searched for. The first derivative, i.e. the gradient, is a local measure of the descent of the surface, while the second derivative (the Hessian) is a measure of the local curvature. More details are given in a subsection on numerical methods.

### Optimal quadrature design

Another dimension in which the described approach can be improved on is the choice of a quadrature. Trapezoid and log-trapezoid rules are the simplest approaches to determine AUC. Their drawbacks were frequently indicated [16, 17]. In the present work not only sampling points are free parameters. Some additional freedom is allowed regarding the choice of a quadrature. This may be done by considering a certain class of quadratures parameterized in a reasonable manner. Here the class of LC methods, as previously introduced by the present author [13], will be considered.

The LC-type quadrature by definition has the form given by Eq. 1, but *w*_*i*_ can now be arbitrary; they do not need to satisfy Eq. 2. *t*_*i*_ are *knots* of the quadrature and *w*_*i*_ are its *weights*.

Surprisingly, many approaches used in pharmacokinetics belong to this class. In particular, linear trapezoidal, hyperbolic trapezoidal [18], Lagrange [19] and spline [4] methods all are of the LC type. The same applies to other popular general methods of numerical analysis, like Newton-Côtes, Gauss-Legendre (GL) or Clenshaw-Curtis (CC) quadratures [20].

Allowing weights vector $\varvec{w}$ as well as knots vector $\varvec{t}$ to be manipulated, results in a final statement for the minimum that should be reached by the optimal method:

$$\begin{aligned} \min _{\varvec{w}, \varvec{t}} \max _{\varvec{\theta }} R_r(\varvec{\theta }) = \min _{\varvec{w}, \varvec{t}} \max _{\varvec{\theta }} E \left[ \left( \frac{\hat{Q} - AUC (\varvec{\theta })}{AUC(\varvec{\theta })} \right) ^2 \right] .\ \end{aligned}$$

Equation 8 remains valid.

In this problem a maximization over $\varvec{\theta }$ is nested within a minimization in both $\varvec{t}$ and $\varvec{w}$. The dimension of search space of outer minimization is twice as large as it is for the optimal trapezoid problem from previous subsection. The optimization task is thus more difficult than in the previous case, and gradient and Hessian of inner maximization, as discussed in the preceding subsection, may prove even more useful.

### Examples chosen for evaluation

Five examples of hypothetical models were analyzed:

1. one-compartment linear model with first-order absorption, single dose;
2. one-compartment linear model with first-order absorption, steady state;
3. two-compartment linear model with iv bolus administration;
4. one-compartment model with iv bolus administration and Michaelis-Menten elimination;
5. one-compartment model with first-order absorption and Michaelis-Menten elimination.

An interval from $t_1=0$ to $t_2=24\mathrm {h}$ was chosen for $AUC$. The dosing interval ($\tau$) for model 2 also matched that interval: $\tau =24\mathrm {h}$. For each model either ranges or fixed values of parameters were assumed. They are given in Table 1 along with the resultant *AUC* range. It is explained in the Appendix why some parameters can be fixed and which of them to choose.

**Table 1:** Model parameter ranges and fixed values

Model	Equation	Parameter($\varvec{\theta }$ or *AUC*)	Range or fixed value
1	$C=A \left( e^{-k_e t}-e^{-k_a t} \right)$	$k_a$	$[(\ln 2) / 4,3 \ln 2]$
		$k_e$	$[(\ln 2) / 12,(\ln 2) / 4]$
		$A=\frac{F D k_a}{V_d \left( k_a-k_e \right) }$	20
		*AUC*	[56, 250]
2	$C= A \left( \frac{e^{-k_e t}}{1 - e^{-k_e \tau }} - \frac{e^{-k_a t}}{1 - e^{-k_a \tau }} \right)$	$k_a$	$[(\ln 2) / 4,3 \ln 2]$
		$k_e$	$[(\ln 2) / 12,(\ln 2) / 4]$
		$A=\frac{F D k_a}{V_d \left( k_a-k_e \right) }$	20
		*AUC*	[58, 337]
3	$C=A_1 e^{-k_1 t}+A_2 e^{-k_2 t}$	$k_1$	$[0.5 \ln 2,6 \ln 2]$
		$k_2$	$[(\ln 2) / 4,(\ln 2) / 24]$
		$$\alpha =A_2/A_1$$	[0.8, 1.25]
		*A*_1_	10
		*AUC*	[50, 245]
4	$\frac{d {C}}{d {t}} = - \frac{V_m C}{K_M+C}$	$K_M$	[2, 20]
		$$V_m$$	[0.2, 1]
		$C_0=C(0)$	10
		*AUC*	[70, 221]
5	$\frac{d {C}}{d {t}} = - \frac{V_m C}{K_M+C}\;+\;A e^{-k_a t}$	$K_M$	[2, 5]
		$$V_m$$	[0.4, 0.7]
		$k_a$	$[(\ln 2)/2,\ln 2]$
		$A=\frac{F D k_a}{V_d}$	5
		*AUC*	[52, 232]

Three levels of coefficient of variation were assumed: 10%, 5% and 0% (no random error). They were combined with three sample sizes chosen for presentation: $n=2$, 4 and 6. This is a range of sample sizes considered, among others, while developing limited sampling strategies. As it will be seen, at greater samples the difference between different approaches becomes less evident.

### Numerical methods

MATLAB 7.11 (R2011b) software (The MathWorks, Inc.) with Minimization Toolbox [21, 22] was used to perform the required computations. A set of M-files written for that purpose is available as supplementary material to this paper. In order to obtain a solution to the minimax problem the ‘fmincon’ procedure from Minimization Toolbox was used on two levels of recursion. This is a general-purpose constrained nonlinear minimization procedure. It contains a variety of optimization algorithms that can be chosen by the user. At outer level (minimization in $\varvec{w}$ and $\varvec{t}$) an *active-set optimization* was chosen, the choice of which implies application of sequential quadratic programming (SQP) algorithm. This algorithm belongs to the *quasi*-Newton family and it can perform better, if derivatives of the objective function are available. It is explained in the Appendix, how the gradient of maximum risk can be computed.

At the inner level (maximization of risk in $\varvec{\theta }$) a *trust-region-reflective* algorithm was preferred. It requires both gradient (first-order derivatives) and Hessian (second-order derivatives) of the objective (in this case the risk function itself) in model parameters.

If a concentration-time dependence and AUC can be expressed in a closed-form by model parameters, then the exact calculation of derivatives imposes no significant difficulty. This is the case with linear compartment models. In the case of one-compartment nonlinear model, with Michaelis-Menten elimination and bolus iv input,*C*(*t*) cannot be expressed in a closed-form, but it may be represented by an implicit function. By theorem on implicit function derivative, differentiation of *C*(*t*) in this case does not introduce true complications either. Moreover, there is a closed-form expression for an AUC given *C*(*t*) at integral limits [17]. However, in order to keep the software as simple as possible, this solution has not actually been used. Conversely, the same solution has been applied to model 4, as it is described just below for model 5.

For the one-compartment nonlinear model, with Michaelis-Menten elimination and first-order input no closed-form solution exists, and a differential equation of the model has to be numerically solved. The MATLAB procedure ‘ode113’, implementing a variable order Adams-Bashforth-Moulton method was used to that purpose (for detailed description of all numerical algorithms used refer to MATLAB documentation [21, 22]). In addition to the required *C*(*t*) values, a differential equation solver can also yield a value of *AUC* along with derivatives of both *C*(*t*) and *AUC*. The necessary details are given in the Appendix.

For each combination of model, sample size and *c*_*v*_ an optimal minimax method was found. Moreover, an optimal trapezoid method and either Gauss-Legendre or Clenshaw-Curtis approximation (whichever performed better) were also found.

The trapezoid method was subject to the following restriction: the last knot always had to be placed at the end of the time interval. On the other hand a linear extrapolation to the time zero was allowed for models with $C_0>0$. This asymmetry was due to the fact that the trapezoid method with a linear extrapolation to $t=\tau$ no longer belongs to the LC class. An extrapolation to $t=0$ does not introduce that problem [13].

### Simulations

In order to evaluate results, for each case 20,000 PK random profiles were simulated. PK parameters were uniformly drawn from their ranges. Based on them concentrations were calculated according to assumed models and Gaussian random noise with assumed *c*_*v*_ was subsequently applied. Using those samples a bias, a root mean squared relative error (RMSRE), minimum and maximum absolute errors and their relative counterparts were estimated.

While the present approach has been developed with mild assumptions on statistical distribution of $\varvec{\theta }$ and $\varvec{\varepsilon }$, for simulation purposes a particular distribution had to be chosen. In order to perform a more demanding evaluation, one may use for $\varvec{\theta }$ a distribution that results in a harder test than normal distribution or log-normal distribution, which are usually applied. A (multidimensional) uniform distribution creates the opportunity to scan a parameter space. It is a common choice in a Monte-Carlo optimum search.

The main rationale for simulations was to investigate those aspects of performance of optimal methods that do not comprise the criteria of optimality. The statistical parameters (RMSRE, bias, etc.) provide simple measures for that purpose. In addition they facilitate a simple check of results. The following inequality is to be expected for any method:

$$\begin{aligned} RMSRE<\sqrt{Obj}, \end{aligned}$$

where $Obj$, the objective, is the maximum risk found at chosen knots and weights. Moreover, in a case without random noise, the maximum observed relative deviance cannot be greater than the square root of the objective unless the optimization failed. This relation may be reversed in the presence of random error.

These inequalities are discussed in detail in the Appendix.

## Results

The results of the optimum method search and the related simulations are compiled in Tables 2, 3, 4, 5, and 6. Footnote labels in the body of Table 2 are referred to in the discussion. They indicate examples of specific behaviour of results.

**Table 2:** Properties and performance of the linear quadrature, optimal in the minimax sense, compared to the optimal trapezoid and GL or CC approaches for Model 1

*n*	$c_v [\%]$	Method	$\sqrt{\mathrm {Objective}}$	Bias	RMSRE	Maximum deviance	Maximum relative deviance
6	10	optimal	4.53E−2^a^	.349	4.34E−2	−36.0^b^	−.1797
		opt. trap.	4.67E−2	−.324	4.48E−2	−35.6	−.1912
		GL	5.79E−2	.741	5.12E−2	38.8	.2203
	5	optimal	2.40E−2	−.392	2.31E−2	−18.6	−.0933
		opt. trap.	2.57E−2	−.769	2.39E−2	−19.8	−.0973
		GL	3.33E−2	.641	2.61E−2	19.5	.1015
	0	optimal	2.03E−4	0.009	1.12E−4	0.03	2.03E−4
		opt. trap.	1.64E−2	−1.033	8.19E−3	−4.08	−1.64E−2
		GL	1.90E−2	0.662	6.46E−3	1.98	1.89E−2
4	10	optimal	5.73E−2	−0.870	5.45E−2	49.2	.2187^c^
		opt. trap.	5.86E−2	−1.230	5.57E−2	46.3	−.2116
		CC	8.84E−2	−3.600	6.81E−2	−47.8	−.2601
	5	optimal	2.80E−2	0.041	2.65E−2	−22.1	.1086
		opt. trap.	3.26E−2	−0.860	2.88E−2	−28.8	−.1187
		CC	6.99E−2	−3.462	4.27E−2	−27.3	−.1696
	0	optimal	2.00E−3	0.003	1.03E−3	0.49	−.0020
		opt. trap.	2.13E−2	0.219	1.07E−3	−4.22	−.0208
		CC	6.26E−2	−3.896	2.96E−3	−6.00	−.0624
2	10	optimal	0.085	−2.23	0.076	−61.1	.2943
		opt. trap.	0.137	−8.42	0.109	−88.8	−.3933
		GL	0.244	13.87	0.143	73.8	.5326
	5	optimal	0.050	−0.049	0.039	27.3	.1626
		opt. trap.	0.116	−6.693	0.073	−51.2	.2537
		GL	0.225	13.948	0.118	47.7	.3716
	0	optimal	0.070	−4.36	0.042	−17.4	−.0698
		opt. trap.	0.108	−6.07	0.056	−26.8	−.1076
		GL	0.218	13.95	0.109	21.7	.2176

^a^objective for the optimal method not significantly less than for the optimal trapezoid

^b^the maximum absolute deviance inferior for the optimal method

^c^the maximum relative deviance inferior for the optimal method

**Table 3:** Properties and performance of the linear quadrature, optimal in the minimax sense, compared to the optimal trapezoid and GL or CC approaches for Model 2

$n$	$c_v[\%]$	Method	$\sqrt{\mathrm {Objective}}$	Bias	RMSRE	Maximum deviance	Maximum relative deviance
6	10	optimal	5.19E−2	1.18	4.85E−2	54.7	−.2273
		opt. trap.	6.12E−2	−1.95	5.11E-2	52.2	−.2287
		GL	5.78E−2	0.65	5.05E−2	56.2	−.2273
	5	optimal	2.27E−2	0.037	2.20E−2	−22.5	.0877
		opt. trap.	2.40E−2	−0.614	2.29E−2	−23.3	.0920
		GL	3.32E−2	0.649	2.61E−2	26.8	.1183
	0	optimal	6.37E−4	0.035	3.65E−4	−0.21	6.37E−4
		opt. trap.	7.80E−3	−0.572	4.31E−3	−2.53	7.78E−3
		GL	1.87E−2	0.675	6.04E−3	1.98	1.86E−2
4	10	optimal	5.41E−2	0.024	5.20E−2	51.3	.2070
		opt. trap.	6.36E−2	−1.963	5.89E−2	−68.0	−.2453
		CC	8.74E−2	−3.517	6.98E−2	66.2	−.2647
	5	optimal	2.95E−2	0.32	2.84E−2	−27.2	−.1115
		opt. trap.	3.30E−2	−1.70	3.14E−2	−32.8	−.1328
		CC	6.86E−2	−3.40	4.60E−2	−32.4	−.1739
	0	optimal	1.94E−3	0.004	9.77E−4	0.64	.0019
		opt. trap.	4.41E−2	−2.702	2.09E−2	−10.84	−.0440
		CC	6.12E−2	−3.341	2.75E−2	−5.99	−.0611
2	10	optimal	0.083	2.16	0.076	103	−.3376
		opt. trap.	0.210	−0.46	0.122	108	.5854
		GL	0.239	13.82	0.137	101	.5536
	5	optimal	0.049	−2.31	0.041	−41.2	−.1815
		opt. trap.	0.190	0.26	0.089	68.9	.3288
		GL	0.219	13.75	0.109	61.3	.3652
	0	optimal	0.026	1.50	0.014	8.3	.0255
		opt. trap.	0.183	0.46	0.076	31.0	.1825
		GL	0.212	13.77	0.043	21.6	.2115

**Table 4:** Properties and performance of the linear quadrature, optimal in the minimax sense, compared to the optimal trapezoid and GL or CC approaches for Model 3

$n$	$c_v [\%]$	Method	$\sqrt{\mathrm {Objective}}$	Bias	RMSRE	Maximum deviance	Maximum relative deviance
6	10	optimal	4.53E-2	0.787	4.30E−2	32.7	.1712
		opt. trap.	6.01E−2	−0.602	4.74E−2	−31.2	.2071
		GL	5.74E−2	−0.329	4.89E−2	−36.9	−.1995
	5	optimal	2.28E−2	0.312	2.22E−2	15.1	.0960
		opt. trap.	3.81E−2	−0.197	2.52E−2	13.8	.1122
		GL	3.32E−2	−0.334	2.48E−2	−15.3	.1114
	0	optimal	1.35E−3	0.027	6.24E−4	0.29	−.0013
		opt. trap.	3.33E−2	−0.439	1.34E−2	3.16	.0328
		GL	1.96E−2	−0.333	5.06E−3	−0.99	−.0190
4	10	optimal	5.47E−2	0.810	5.30E−2	−44.3	−.2187
		opt. trap.	7.80E−2	−1.105	5.93E−2	−40.4	.2808
		CC	9.17E−2	1.937	6.49E−2	40.2	.2897
	5	optimal	2.84E−2	0.084	2.79E−2	20.1	.1083
		opt. trap.	5.32E−2	−0.986	3.29E−2	−21.0	.1346
		CC	6.69E−2	1.849	3.27E−2	20.7	.1616
	0	optimal	5.72E−3	0.024	2.47E−3	1.22	.0057
		opt. trap.	4.18E−2	−0.833	1.59E−2	4.44	.0417
		CC	5.62E−2	1.890	2.21E−2	3.34	.0551
2	10	optimal	0.104	2.573	0.083	57.5	.3339
		opt. trap.	0.120	−0.503	0.095	−61.1	.4148
		GL	0.227	−8.866	0.122	−55.2	−.4542
	5	optimal	0.075	1.948	0.046	33.6	.1917
		opt. trap.	0.085	−0.130	0.052	−28.6	.2180
		GL	0.218	3.908	0.104	−29.3	.3566
	0	optimal	0.062	−1.174	0.021	6.76	.0603
		opt. trap.	0.070	−0.042	0.026	−8.22	.0663
		GL	0.215	−8.841	0.097	−13.97	−.2121

**Table 5:** Properties and performance of the linear quadrature, optimal in the minimax sense, compared to the optimal trapezoid and GL or CC approaches for Model 4

$n$	$c_v[\%]$	Method	$\sqrt{\mathrm {Objective}}$	Bias	RMSRE	Maximum deviance	Maximum relative deviance
6	10	optimal	4.52E−2	−1.117	4.39E−2	37.1	.1821
		opt. trap.	5.76E−2	0.458	4.28E−2	33.2	.1982
		GL	5.61E−2	0.073	4.50E−2	−32.6	.1987
	5	optimal	2.28E−2	−.5892	2.22E−2	−17.7	−.0937
		opt. trap.	3.39E−2	.2958	2.17E−2	16.4	.1040
		GL	2.81E−2	.0079	2.25E−2	−16.7	.0942
	0	optimal	5.05E−5	4.16E−3	2.40E−5	.0058	−4.63E−5
		opt. trap.	4.01E−3	−5.84E−2	6.66E−4	.2843	−3.04E−3
		CC	5.63E−5	−2.40E−5	2.18E−6	−.0039	−5.15E−5
4	10	optimal	6.54E−2	1.779	5.69E−2	42.4	.2615
		opt. trap.	8.60E−2	0.488	5.59E−2	41.3	.2950
		GL	6.79E−2	0.028	5.40E−2	42.2	.2429
	5	optimal	3.32E−2	1.211	2.83E−2	−20.5	−.1076
		opt. trap.	4.43E−2	0.143	3.11E−2	19.1	.1312
		GL	3.39E−2	0.046	2.69E−2	−23.0	−.1095
	0	optimal	3.63E−4	3.82E−2	2.29E−4	.0614	−3.59E−4
		opt. trap.	6.43E−3	−1.50E−1	1.63E−3	−.6145	−6.30E−3
		GL	6.54E−4	−2.96E−4	2.58E−5	−.0404	−5.16E−4
2	10	optimal	0.089	6.001	8.35E−2	66.7	.3400
		opt. trap.	0.103	−0.173	8.72E−2	−68.1	.3920
		GL	0.110	0.088	7.37E−2	55.8	.3252
	5	optimal	4.52E−2	3.778	4.37E−2	33.1	.1683
		opt. trap.	5.56E−2	−0.054	4.36E−2	−31.3	−.1739
		GL	6.56E−2	0.087	3.72E−2	−28.3	.1598
	0	optimal	7.01E−3	0.882	5.55E−3	1.43	.0070
		opt. trap.	2.40E−2	−0.065	2.70E−3	2.18	.0238
		GL	4.11E−2	0.041	2.04E−3	2.77	.0393

**Table 6:** Properties and performance of the linear quadrature, optimal in the minimax sense, compared to the optimal trapezoid and GL or CC approaches for Model 5

$n$	$c_v [\%]$	Method	$\sqrt{\mathrm {Objective}}$	Bias	RMSRE	Maximum deviance	Maximum relative deviance
6	10	optimal	4.40E−2	−0.202	4.22E−2	−28.3	.1559
		opt. trap.	4.52E−2	−1.567	4.45E−2	−31.1	−.1635
		GL	5.54E−2	−0.030	4.85E−2	30.3	.1881
	5	optimal	2.24E−2	−0.251	2.16E−2	−13.5	−.0876
		opt. trap.	2.49E−2	−1.229	2.36E−2	−15.2	−.0956
		GL	2.77E−2	−0.019	2.40E−2	15.5	.1110
	0	optimal	4.67E−5	−0.0004	2.21E−5	9.43E−2	4.65E−5
		opt. trap.	1.47E−2	−1.3228	1.05E−2	−2.827	1.47E−2
		GL	5.03E−4	0.0133	1.45E−4	0.026	4.90E−4
4	10	optimal	5.39E−2	0.325	5.14E−2	−39.2	.2087
		opt. trap.	6.00E−2	−2.808	5.80E−2	−38.9	−.2236
		GL	6.64E−2	0.357	5.73E−2	−38.0	.2132
	5	optimal	2.73E−2	0.641	2.65E−2	19.1	.1082
		opt. trap.	3.69E−2	−2.358	3.32E−2	−21.6	−.1324
		GL	3.54E−2	0.433	2.92E−2	−22.3	.1142
	0	optimal	6.87E−4	−0.021	3.76E−4	0.14	6.85E−4
		opt. trap.	2.61E−2	−2.271	1.80E−2	−5.26	−2.60E−2
		GL	1.42E−2	0.466	4.90E−3	0.75	1.40E−2
2	10	optimal	0.078	0.64	0.074	−50.5	.3123
		opt. trap.	0.144	−11.78	0.120	−66.4	.4163
		GL	0.203	10.42	0.123	58.9	.4326
	5	optimal	0.045	1.37	0.039	30.3	.1656
		opt. trap.	0.128	−11.87	0.101	−48.9	−.2419
		GL	0.180	10.39	0.099	40.0	.2712
	0	optimal	4.02E−3	0.12	2.17E−3	−0.88	.0040
		opt. trap.	1.23E−1	−11.96	9.41E−2	−22.93	−.1220
		GL	1.72E−1	10.41	8.96E−2	12.10	.1633

**Fig. 1:**
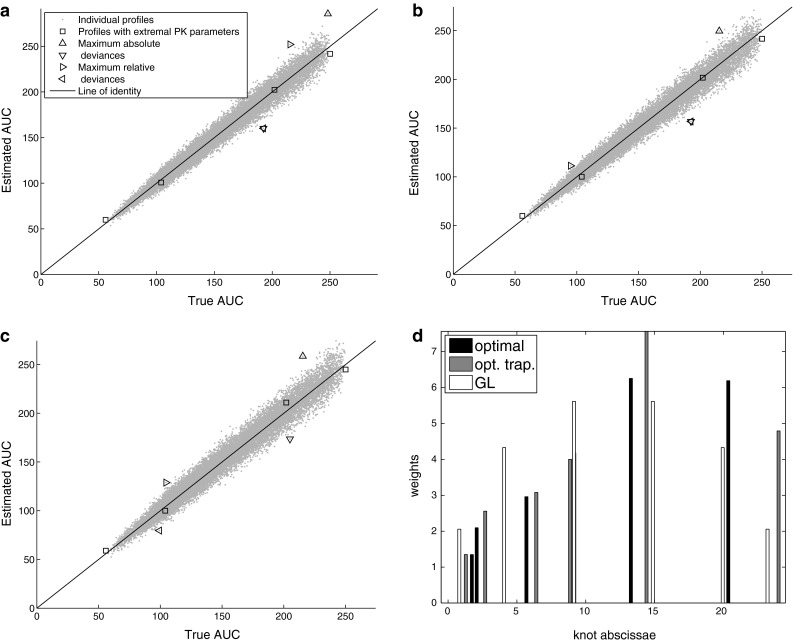
Performance of the investigated methods for Model 1 with *n*=6 and $c_v=0.10$. Panels depict true vs estimated AUC by (**a**) optimal, (**b**) optimal trapezoid, and (**c**) Gauss-Legendre methods. Panel (**d**) displays knots and weights of these methods (one bar of optimal method is hidden behind bars of other methods)

**Fig. 2:**
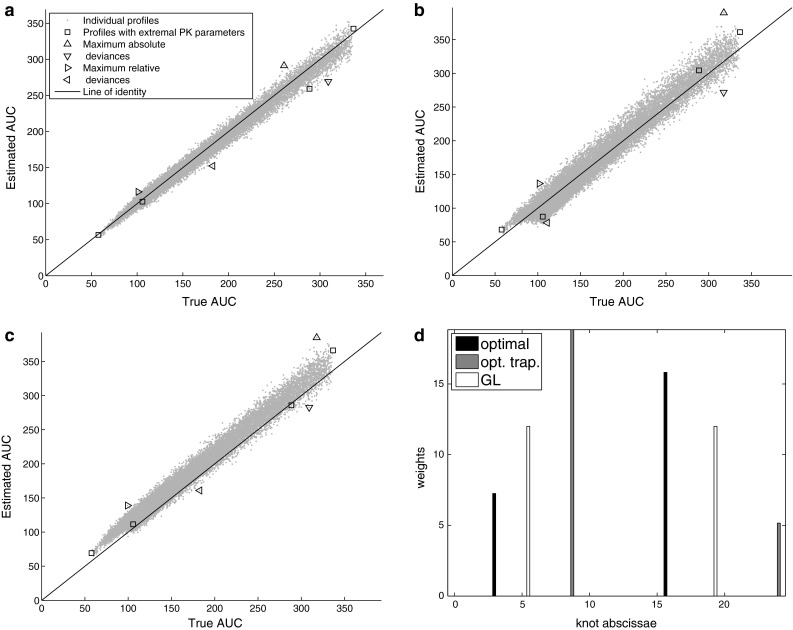
Performance of the investigated methods for Model 2 with *n*=2 and $c_v=0.05$. Panels depict true vs estimated AUC by (**a**) optimal, (**b**) optimal trapezoid, and (**c**) Gauss-Legendre methods. Panel (**d**) displays knots and weights of these methods

**Fig. 3:**
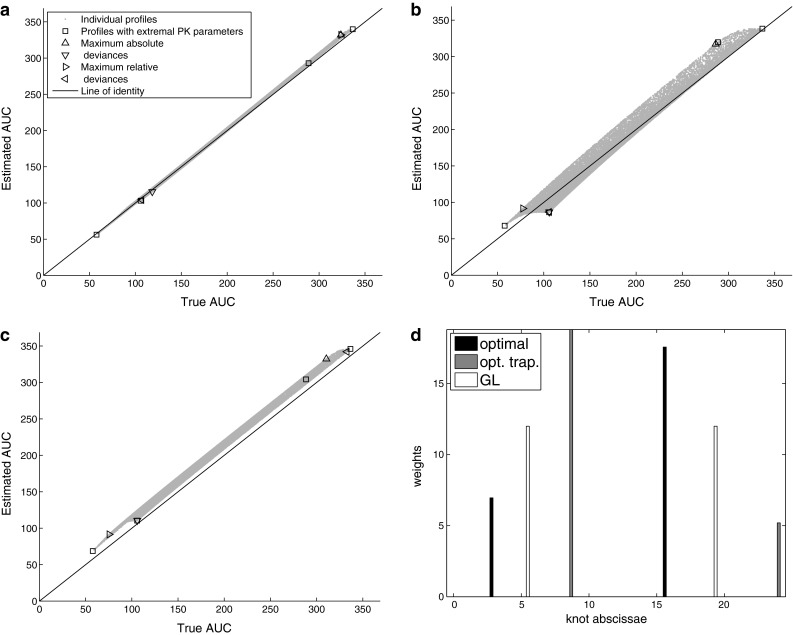
Performance of the investigated methods for Model 2 with n=2 and $c_v=0$ (no random error). Panels depict true vs estimated AUC by (**a**) optimal, (**b**) optimal trapezoid, and (**c**) Gauss-Legendre methods. Panel (**d**) displays knots and weights of these methods

A few representative plots showing the quality of the predictions can be found in Figs. 1, 2, and 3. These plots show each simulated case as a small gray dot. Its abscissa equals to the true AUC value, i.e. calculated based on PK parameters values assumed in the simulation, and its ordinate represents the result of estimation. As it is quite common that maximum risk is reached at the extremal values of some or all parameters the special points simulated for these extremal values are indicated by open square symbols ($\Box$). Open triangles indicate those points at which the maximum estimation error appeared: $\triangleleft$ and $\triangleright$ are for maximum relative under- and overestimates, respectively; while $\triangledown$ and $\vartriangle$ are for maximum absolute under- and overestimated results in a plot. In these figures, on separate plots, the knots and weights of all three methods are also depicted. The position of each bar is that of a knot while its height represents a value of weight.

**Fig. 4:**
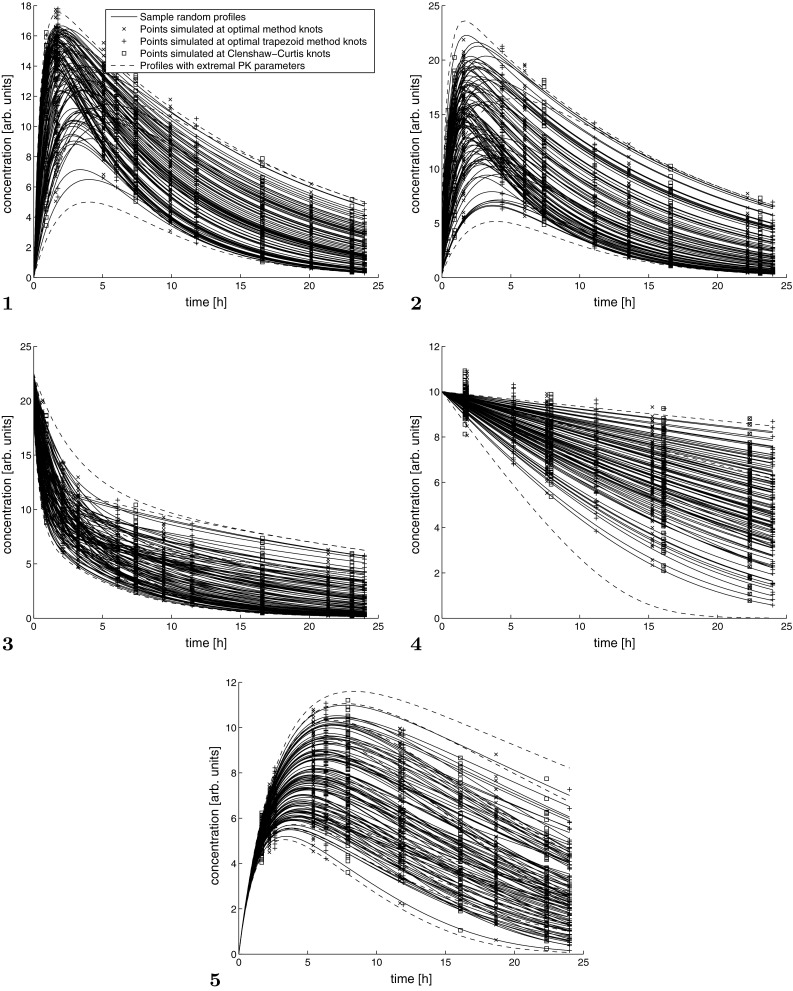
Spaghetti plots for $n=4$ and $c_v=0.05$ across models **1–5**

**Fig. 5:**
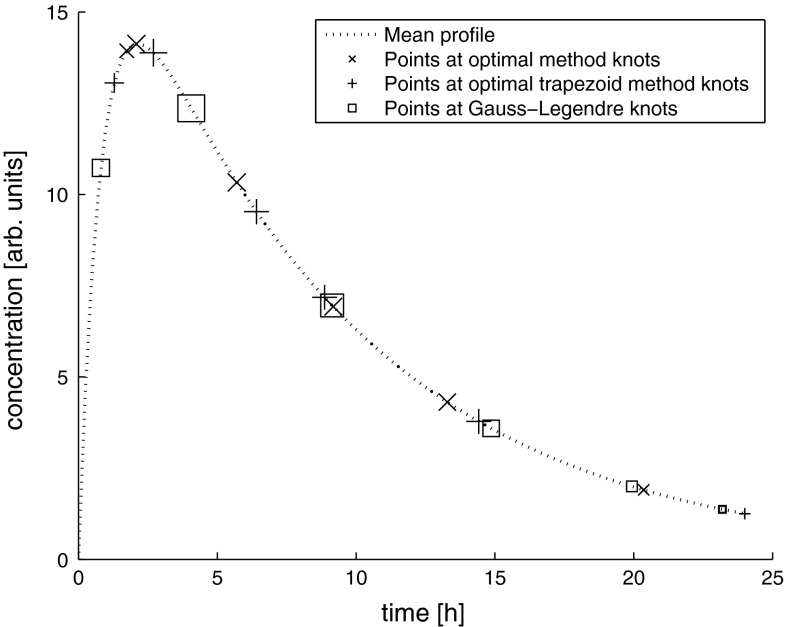
Mean profile for Model 1. Area covered by each symbol is proportional to a contribution of a concentration value at given knot into AUC value estimated by the corresponding method

Figure 4 contains sample spaghetti plots for all models at $c_v=0.1$ and $n=4$. Each 200th PK profile (of 20,000) is shown along with the corresponding concentrations measured at knots of investigated methods. Yet another manner of comparison of methods is displayed in Fig. 5. It contains a “mean” profile for Model 1, i.e. a profile simulated at midpoint values of PK parameters. The symbols are plotted at knots of the corresponding methods. The area covered by each symbol is proportional to its contribution to the total AUC.

## Discussion

The objective for the optimal method was not always significantly less than for the optimal trapezoid (Table 2, footnote $^{\mathrm{a}}$). For a richer sampling ($n=6$) and higher variability ($c_v=0.1$) the maximum risk of all methods was comparable: the objective of the optimal method was never lower than 30% of the worst method’s objective. On the other hand for $c_v=0$ the objectives differed to even more than five orders of magnitude. Differences also appeared to be more pronounced with a decrease in the sample size. It can also be confirmed by inspection of the Figures: patterns on Fig. 1 (richer sampling, higher variability) are rather similar across all three methods, while on Fig. 2 (very sparse sampling) and Fig. 3 (no variability, in addition) patterns are quite different.

Several maximum absolute deviances appeared to be inferior for the optimal method (Table 2, footnote $^{\mathrm{b}}$). Furthermore, it happened that the maximum relative deviance observed was worse for an optimal method than for one of the other methods (Table 2, footnote $^{\mathrm{c}}$). There was no contradiction in this, since the neighbourhood of such $\varvec{\theta }$, at which other methods would perform worse than the optimal one, might simply has been missing in the simulation.

There was no clear superiority of either bias or RMSRE for the optimal method in comparison to other methods. Also, one cannot clearly indicate which one of the alternative methods had lower risk across the investigated models.

Even for as large a sample as $n=6$, the maximum relative deviance was of order of 20% for any method at $c_v=0.10$. Assuming the deviance of 20% is at the limit of usefulness, it could be provided in most cases also for $n=2$ and $c_v=0.05$ using the optimal method with relative risk.

Inequality 18 discussed in the Appendix was satisfied for samples with $c_v>0$, confirming that random errors outweigh quadrature errors. For samples with $c_v=0$ inequality 17 was satisfied, indicating successful optimization.

The precise definition of the optimal AUC estimation method depends on the choice of risk function, considered class of quadratures, and the interpretation of the PK parameters vector (Bayesian or minimax estimation). The present paper contains an analysis of the specific combination of these factors: quadratic loss function, LC quadratures, and minimax estimator. It was demonstrated how much progress may be made by transition from the simple trapezoid method to the optimal LC quadrature in the above sense.

That LC-quadratures are distinguished may be argued as follows: In the framework of linear pharmacokinetics they guarantee linearity of the AUC estimates. With non-linear quadratures the calculation of risk function can be quite difficult and it may require full knowledge of probability distribution of random errors. Also, an application of numerical integration algorithms, including Monte-Carlo, might be necessary. Likewise, the minimax approach enables more general treatment than the Bayesian framework, since the latter depends on the prior distribution of model parameters, which is not necessarily known. On the other hand, in order to successfully apply the present method, the appropriate PK model has to be identified beforehand and the range of certain PK parameters as well as $c_v$ should be estimated.

Thus prior investigation of the drug on the target population is required. Along with observed clearer superiority on small samples it suggests that developing of limited sampling strategies may constitute an area of application for this approach.

**Fig. 6:**
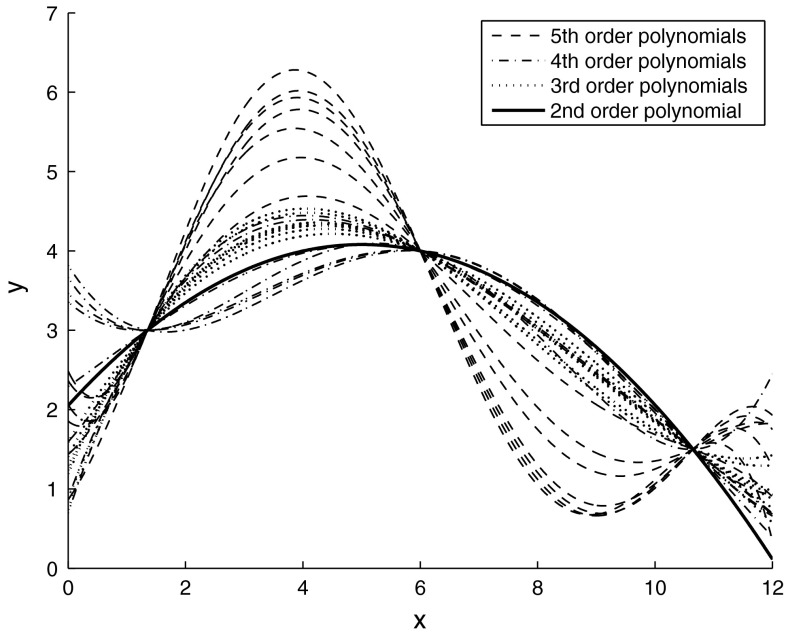
Sample polynomials passing through three given points. All polynomials have the same integral in the interval [0, 2]. Only a square polynomial is uniquely determined

At this point one may wonder, why not simply fit the model to the data and use obtained PK parameters to calculate AUC? Although it is possible in principle, it is an indirect solution, and as such it does not need to be optimal. To illustrate this the following analogy can be developed:

Gauss-Legendre quadrature of order *n* (i.e. having *n* knots) is exact for any polynomial of an order up to $2n-1$. For instance, if a polynomial value is known at three properly chosen knots, this polynomial can be exactly integrated, provided its order is 5 or less. But no polynomial coefficients can be determined for polynomials of an order $\ge 3$. In fact, there is a continuum of polynomials, passing through those given points, with the same integral; a few of them are depicted in Fig. 6.

While it is believed that the case discussed herein is an important one, it is by no means the only one that deserves analysis. In particular, non-linear quadratures are certainly worth investigation, despite the difficulties indicated above, as they are more general. The widely used log-trapezoid rule is a very simple instance of such a quadrature.

The AUC in the finite interval is more appropriate to the steady state. Amisaki gives an important insight into the problem of integration in the infinite interval [23]; this topic also appears to be worth further analysis.

## Conclusions

Optimal linear minimax estimator of AUC in the finite interval can be effectively constructed for PK models, regardless of whether they are given by an explicit *C*(*t*) relationship or defined by the differential equations. The developed method may also be applied in other disciplines, where estimation of integrals from sparse and noisy data is essential.

The optimal method may appear significantly better than other considered methods for low variability samples. On the other hand, for larger samples with higher variability it is less advantageous and it may be replaced by the simpler method. In particular, GL and CC algorithms may then be considered, since their weights and knots do not depend on the model nor the range of model parameters.

There is no optimal AUC estimator in the universal sense, but what is meant by 'optimal' depends on so many factors that it appears that the answer to the question in the title should be positive.

The benefits of the minimax estimator with the LC quadrature and constant *c*_*v*_ may be summarized as follows:

1. To obtain the estimator one does not need to know PK parameters distribution; no covariance matrix is necessary. Only a reasonable range of parameters should be determined.
2. Detailed knowledge on experimental error is also unimportant. Zero mean and constant *c*_*v*_ conditions suffice.
3. A construction of the estimator does not involve multidimensional integrals and their numerical approximations.
4. A constant *c*_*v*_ condition with a relative risk simplifies the estimator construction process, even for nonlinear models. This is discussed in the first subsection of the Appendix.
5. In a sense, this estimator is more conservative than the Bayesian approach, since it minimizes an error in the worst possible scenario and not in an average situation as does the Bayesian approach.

As a closing remark a comment on the methodology being used for elaborating limited-sampling strategy for a number of drugs [6, 8–10] can be given. Formally, the applied quadrature differs from the LC type only by an additional constant term (the intercept), but the interpretation is quite different. Linear regression is postulated between AUC and concentrations measured at knots. The weights have the meaning of multiple regression coefficients. It might appear, that some kind of maximum likelihood estimator of AUC is constructed in this way. Note however, that postulate of linear dependency is not necessarily true in PK applications. The present paper also uses linear quadrature, but *does not assume* it is exact in the absence of random errors, as the linear regression approach does.

### Electronic supplementary material

Below is the link to the electronic supplementary material.

Supplementary material 1 (PDF 33084 kb)

Supplementary material 2 (ZIP 38 kb)

## Appendix

### Reducing the search space

#### General considerations

Consider two parameter vectors, $\varvec{\theta }$ and $\varvec{\theta }'$, both in the assumed parameter range, such that there exists $\kappa$ so that for any *t*

9 $$\begin{aligned} C(t,\varvec{\theta }')=\kappa C(t,\varvec{\theta }). \end{aligned}$$

Then, in the case of constant *c*_*v*_, $R(\varvec{\theta }')=\kappa ^2 R(\varvec{\theta })$ and $R_r(\varvec{\theta }')=R_r(\varvec{\theta })$.

Thus, if there exists a subset $\varTheta '$, of the parameter range $\varTheta$, such that for any $\varvec{\theta }$ in $\varTheta$ there exists $\varvec{\theta }'$ in $\varTheta '$ so that Eq. 9 holds with $\kappa \ge 1$, then the search space $\varTheta$ can be reduced to $\varTheta '$.

From practical reasons $\varTheta$ is chosen as a multidimensional interval (or combination of such intervals):

$$\begin{aligned} \varTheta = \left\{ \varvec{\theta }: \theta _{min,i} \le \theta _i \le \theta _{max,i}, i= 1 \dots \dim (\varvec{\theta }) \right\} \end{aligned}$$

If $\varTheta '$ can be chosen so that for some *i*, $\theta _i$ is fixed, then a dimension of search space will be reduced. This may significantly decrease the computing time.

### Application to PK models

In the case of linear PK models Eq. 9 is satisfied automatically. For models 1 and 2 let the parameter range be chosen as

$$\begin{aligned} \varTheta =[k_{a\;min},k_{a\; max}] \times [k_{e\; min}, k_{e\; max}] \times [A_{min},A_{max}] \end{aligned}$$

and $$\varTheta '$$ as

$$\begin{aligned} \varTheta '=[k_{a\; min},k_{a\; max}] \times [k_{e\; min}, k_{e\; max}] \times \{A_{max}\}. \end{aligned}$$

For model 3 the choices might be

$$\begin{aligned} \varTheta =&[k_{1\;min},k_{1\; max}] \times [k_{2\; min}, k_{2\; max}] \times [A_{\min },A_{max}] \\&\times [p_{min},p_{max}] \end{aligned}$$

and

$$\begin{aligned} \varTheta '=&[k_{1\;min},k_{1\; max}] \times [k_{2\; min}, k_{2\; max}] \times \{A_{max}\} \\&\times [p_{min},p_{max}]. \end{aligned}$$

For model 4 one can write

$$\begin{aligned} \frac{d {(\kappa C)}}{d {t}} = - \kappa \frac{V_m C}{K_M+C} = - \frac{(\kappa V_m) (\kappa C)}{\kappa K_M + \kappa C}. \end{aligned}$$

This implies that if $C(t)$ is a solution of model equation with parameters $K_M$ and $V_m$ and initial condition $C(0)=C_0$, then $\kappa C(t)$ is a solution of the same equation with model parameters (including initial concentration) multiplied by $\kappa$. It may be viewed as a result of change in mass unit by factor $\kappa$. If one chooses $\varTheta '$ as

$$\begin{aligned} \varTheta '=&[\lambda K_{M\;min},K_{M\;max}]\times [\lambda V_{m\;min},V_{m\;max}] \\&\times \{C_{0\;max}\} \end{aligned}$$

where $\lambda =C_{0\;min}/C_{0\;max}$, then the choice for $\varTheta$ might be

$$\begin{aligned} \varTheta =&\varTheta ' \cup [K_{M\;min},K_{M\;max}]\times [V_{m\;min},V_{m\;max}] \\&\times [C_{0\;min},C_{0\;max}]. \end{aligned}$$

Finally, for model 5 one obtains

$$\begin{aligned} \varTheta '=&[\lambda K_{M\;min},K_{M\;max}]\times [\lambda V_{m\;min},V_{m\;max}] \\&\times [k_{a\;min},k_{a\; max}] \times \{A_{max}\} \end{aligned}$$

and

$$\begin{aligned} \varTheta =\varTheta ' \cup&[K_{M\;min},K_{M\;max}]\times [V_{m\;min},V_{m\;max}] \\&\times [k_{a\;min},k_{a\; max}]\times [A_{min},A_{max}]. \end{aligned}$$

### Outer objective derivative derivation

Let $M(\varvec{\omega }) = \max _{\varvec{\theta }} R(\varvec{\theta },\varvec{\omega })$, where $\varvec{\omega }$ is a vector combined from $\varvec{t}$ and $\varvec{w}$. The $\varvec{\theta }^{(m)}$ that maximizes $R$ clearly depends on $\varvec{\omega }$, so one can write:

$$\begin{aligned} M(\varvec{\omega }) = R \left( \varvec{\theta }^{(m)} (\varvec{\omega }), \varvec{\omega }\right) \end{aligned}$$

Thus

$$\begin{aligned} \frac{d {M(\varvec{\omega })}}{d {\omega _k}} = \sum _{i=1}^{dim(\varvec{\theta })} \frac{\partial {R}}{\partial {\theta _i^{(m)}}} \frac{d {\theta _i ^{(m)}}}{d {\omega _k}} \;+\; \frac{\partial {R}}{\partial {\omega _k}} \end{aligned}$$

If at the maximizer $\frac{\partial {R}}{\partial {\theta _i ^{(m)}}} \ne 0$, it implies that $\theta _i^{(m)}$ must be at one of its bounds. Assuming that $\frac{\partial {R}}{\partial {\theta _i ^{(m)}}}$ is continuous in $\varvec{\omega }$, it will remain non-zero after an infinitesimal change in $\omega _k$ and therefore the maximizer will remain at its bound. It means that $\frac{\partial {\theta _i ^{(m)}}}{\partial {\omega _k}} = 0$. A maximizer may also be in the interior of $\varTheta$ implying that $\frac{\partial {R}}{\partial {\theta _i ^{(m)}}} = 0$. In any case

$$\begin{aligned} \frac{\partial {R}}{\partial {\theta _i^{(m)}}} \frac{\partial {\theta _i ^{(m)}}}{\partial {\omega _k}} = 0 \end{aligned}$$

and

$$\begin{aligned} \frac{d {M(\varvec{\omega })}}{d {\omega _k}} = \frac{\partial {R}}{\partial {\omega _k}} \end{aligned}$$

### Treatment of models defined by differential equations

In order to obtain $AUC$ along with $C(t)$ as well as their derivatives in parameters the model differential equation

10 $$\begin{aligned} \frac{d {C(t,\varvec{\theta })}}{d {t}} = f \left( C \left( t, \varvec{\theta }\right) ,\varvec{\theta },t \right) \end{aligned}$$

has to be supplemented with additional equations, the first of them being:

11 $$\begin{aligned} \frac{d {AUC(t, \varvec{\theta })}}{d {t}} = C(t,\varvec{\theta }), \end{aligned}$$

where $AUC(t,\varvec{\theta })$ is calculated in the time interval from 0 to $t$.

Define:

$$\begin{aligned} g_i(t,\varvec{\theta })&= \frac{d {C(t,\varvec{\theta })}}{d {\theta _i}}\\ G_i(t,\varvec{\theta })&= \frac{d {AUC(t,\varvec{\theta })}}{d {\theta _i}}\\ h_{ij}(t,\varvec{\theta })&= \frac{d {^2 C(t,\varvec{\theta })}}{d {{\theta _i} d {\theta _j} }}\\ H_{ij}(t,\varvec{\theta })&= \frac{d {^2 AUC(t,\varvec{\theta })}}{d {{\theta _i} d {\theta _j} }} \end{aligned}$$

Then

$$\begin{aligned} \frac{d {g_i}}{d {t}}&= \frac{d {}}{d {\theta _i}} {\frac{d {C}}{d {t}}} = \frac{d {}}{d {\theta _i}} f \left( C \left( t, \varvec{\theta }\right) ,\varvec{\theta },t \right) \\&= \frac{\partial {f}}{\partial {C}} \frac{d {C}}{d {\theta _i}} + \frac{\partial {f}}{\partial {\theta _i}} \end{aligned}$$

what may be written as the subsequent equations:

12 $$\begin{aligned} \frac{d {g_i}}{d {t}} = \frac{\partial {f}}{\partial {C}} g_i + \frac{\partial {f}}{\partial {\theta _i}} \end{aligned}$$

and, in the similar way:

13 $$\begin{aligned} \frac{d {G_i}}{d {t}} = g_i \end{aligned}$$

Finally, for the Hessian one obtains:

$$\begin{aligned} \frac{d {}}{d {t}} \frac{d {^2 C}}{d {\theta _i d\theta _j}}= \frac{d {^2 }}{d {\theta _i d\theta _j}} \frac{d {C}}{d {t}} = \frac{d {^2 }}{d {\theta _i d\theta _j}} f \left( C \left( t, \varvec{\theta }\right) ,\varvec{\theta },t \right) \end{aligned}$$

which yields the following equations:

14 $$\begin{aligned} \frac{d {h_{ij}}}{d {t}}&= \frac{\partial {^2 f}}{\partial {C^2}} g_i g_j + \frac{\partial {^2 f}}{\partial {C \partial {\theta _i}}} g_j + \frac{\partial {^2 f}}{\partial {C \partial {\theta _j}}} g_i + \nonumber \\&+ \frac{\partial {f}}{\partial {C}} h_{ij} + \frac{\partial {^2 f}}{\partial {\theta _i \partial \theta _j}} \end{aligned}$$

and

15 $$\begin{aligned} \frac{d {H_{ij}}}{d {t}} = h_{ij} \end{aligned}$$

The differential Eqs. (10 – 15) with initial conditions:

$$\begin{aligned} C(0,\varvec{\theta })&= C_0(\varvec{\theta }),\\ AUC(0,\varvec{\theta })&= 0,\\ g_i(0,\varvec{\theta })&= \frac{\partial {C_0}}{\partial {\theta _i}} ,\\ G_i(0,\varvec{\theta })&= 0 ,\\ h_{ij}(0,\varvec{\theta })&= \frac{\partial {^2 C_0}}{\partial {\theta _i d \theta _j}},\\ H_{ij}(0,\varvec{\theta })&= 0, \end{aligned}$$

if solved, form the complete data needed by the optimization procedure.

In the case of iv administration, if $C_0$ is chosen as a model parameter (along with $K_M$ and $V_m$), so that $\varvec{\theta }=(K_M,V_m,C_0)$ then

$$\begin{aligned} f(C,\varvec{\theta },t)=-\frac{V_m C}{K_M + C} \end{aligned}$$

and

$$\begin{aligned} \frac{\partial {C_0}}{\partial {\varvec{\theta }}} = (0,0,1) \end{aligned}$$

For the first-order absorption with no initial concentration $\varvec{\theta }=(K_M,V_m,k_a,A)$ where $A=F D k_a / V_d$

$$\begin{aligned} f(C,\varvec{\theta },t)=-\frac{V_m C}{K_M + C}+A \exp \left( -k_a t \right) \end{aligned}$$

and

$$\begin{aligned} \frac{\partial {C_0}}{\partial {\varvec{\theta }}} = (0,0,0,0) \end{aligned}$$

### Inequalities

In practice, the expectation of a random variate is always less than its maximal possible value. In most cases it should also be less than the maximum value observed, even for a sample of moderate size. Thus the following inequality must hold:

16 $$\begin{aligned} E_{\varvec{\theta }} R_r(\varvec{\theta })<\max _{\varvec{\theta }} R_r(\varvec{\theta }) \end{aligned}$$

and also, almost certainly:

17 $$\begin{aligned} \max _{\varvec{\theta }\;sample}R_r(\varvec{\theta })&=\max _{\varvec{\theta }\;sample}E_{\varvec{\varepsilon }} L_r(\hat{Q},\varvec{\theta })\nonumber \\ {}&\le \max _{\begin{array}{c} \varvec{\theta }\;sample \\ \varvec{\varepsilon }\;sample \end{array}} L_r(\hat{Q},\varvec{\theta }). \end{aligned}$$

On the other hand, randomly drawn vectors $\varvec{\theta }$ may miss the maximizer:

18 $$\begin{aligned} \max _{\varvec{\theta }}R_r(\varvec{\theta }) \ge \max _{\varvec{\theta }\;sample}R_r(\varvec{\theta }). \end{aligned}$$

$MSRE$ estimates an expectation of the risk and for a large sample it should provide a good approximation. On average, inequality 16 should also hold for expectation replaced by $MSRE$. The relation between objective and maximum observed loss is more complicated, because inequalities 17 and 18 work in opposite directions. If the coefficient of variation $c_v$ is very small, or there is no random error at all, the inequality in 17 may be replaced by an equal sign and inequality 18 becomes important. In a more realistic situation and with a sample large enough one may expect that random error will dominate the quadrature error, as Katz and D’Argenio pointed out. Then inequality 17 should prevail.

Taking square roots of these inequalities one may summarize them as

19 $$\begin{aligned} RMSRE < \sqrt{Obj(\varvec{\theta }^{(m)})}<\max _{\begin{array}{c} \varvec{\theta }\;sample \\ \varvec{\varepsilon }\;sample \end{array}}\frac{|AUC-\hat{Q}|}{AUC} \end{aligned}$$

unless the $c_v$ is 0 or very small, in which case the second inequality sign may be reversed.

### Supplementary material

Additional documentation to this paper is available as electronic supplementary material. It consists of a set of Matlab M-files implementing minimax optimization, and plots of all simulations for Model 1. There are also several sample job configuration files used by a Matlab code. Interested readers may modify the Matlab code and/or configuration files to develop their own models.
