# Supplementary material for: Searching for an optimal AUC estimation method: a never-ending task?
Source: J Pharmacokinet Pharmacodyn. 2014 Oct 15;41(6):655–73. doi: 10.1007/s10928-014-9392-y (PMC4225057; doi:10.1007/s10928-014-9392-y)
Supplement: Supplementary file 1 — Supplementary material 1 (PDF 33084 kb) [file 10928_2014_9392_MOESM1_ESM.pdf]

All Figures for Model 1

Supplementary material to a paper entitled: Searching for an optimal AUC estimation method – a never-ending task? by Wojciech Jawień, Jagiellonian University in Kraków, Poland.

This file contains plots for each combination of sample size and c_v for Model 1. Color version of spaghetti plots for $n = 4$ and $c_v = 5\%$ across all models is also included.

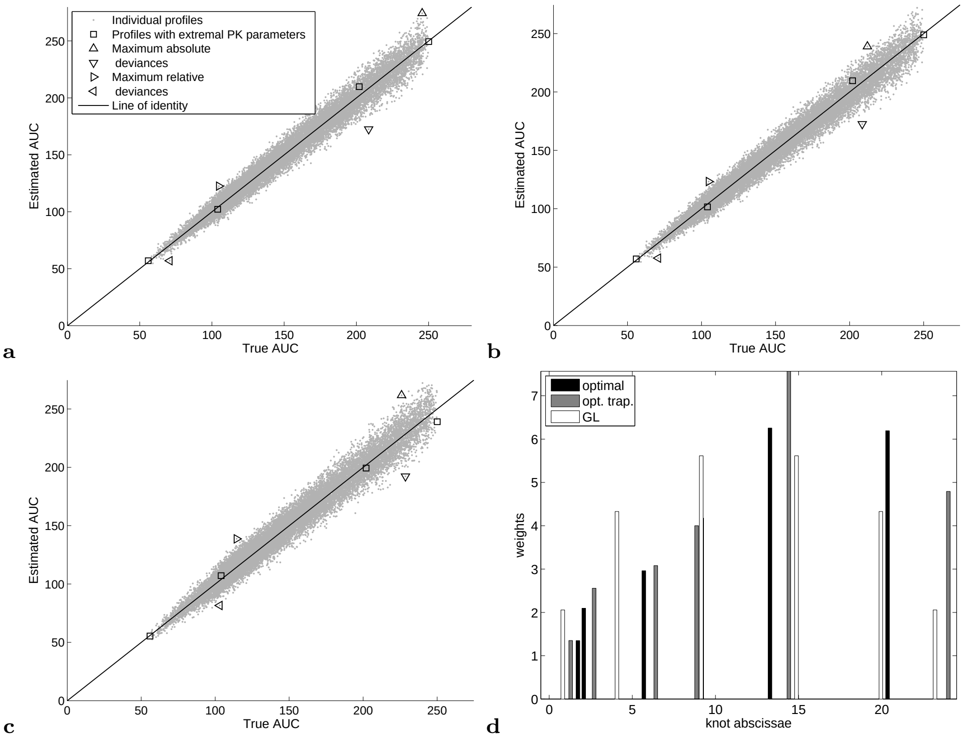

Fig. 1 Performance of the investigated methods for Model 1 with the relative risk,n=6 and c_v=0.10. **a-c**: true vs estimated AUC by optimal, optimal trapezoid and GL or CC method, respectively; **d**: knots and weights of these methods.

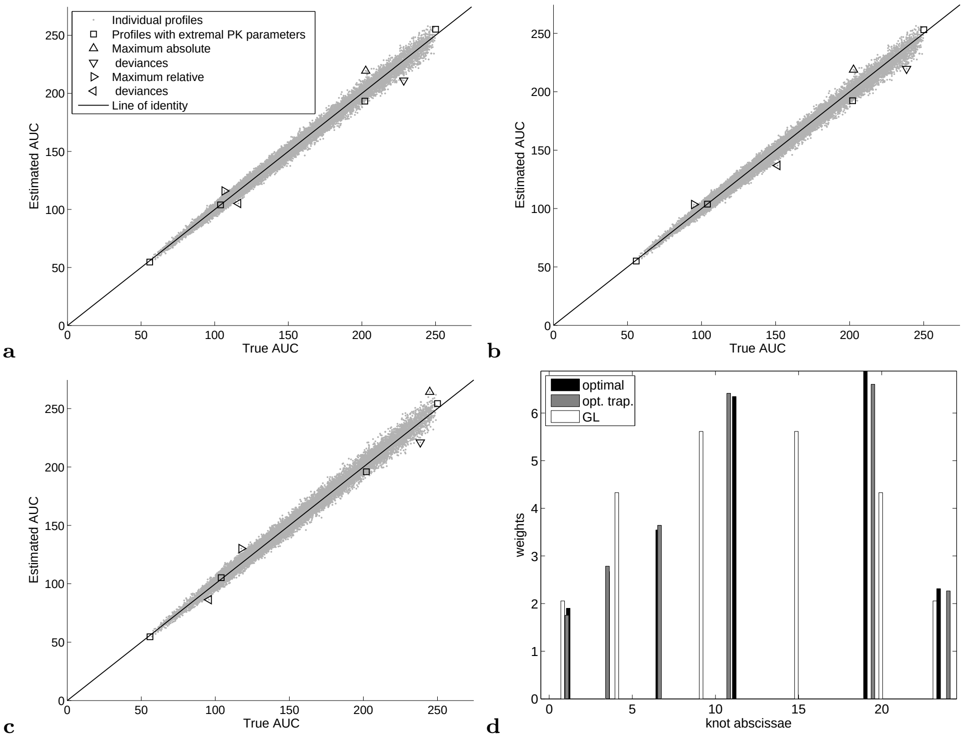

Fig. 2 Performance of the investigated methods for Model 1 with the relative risk,n=6 and c_v=0.05. **a-c**: true vs estimated AUC by optimal, optimal trapezoid and GL or CC method, respectively; **d**: knots and weights of these methods.

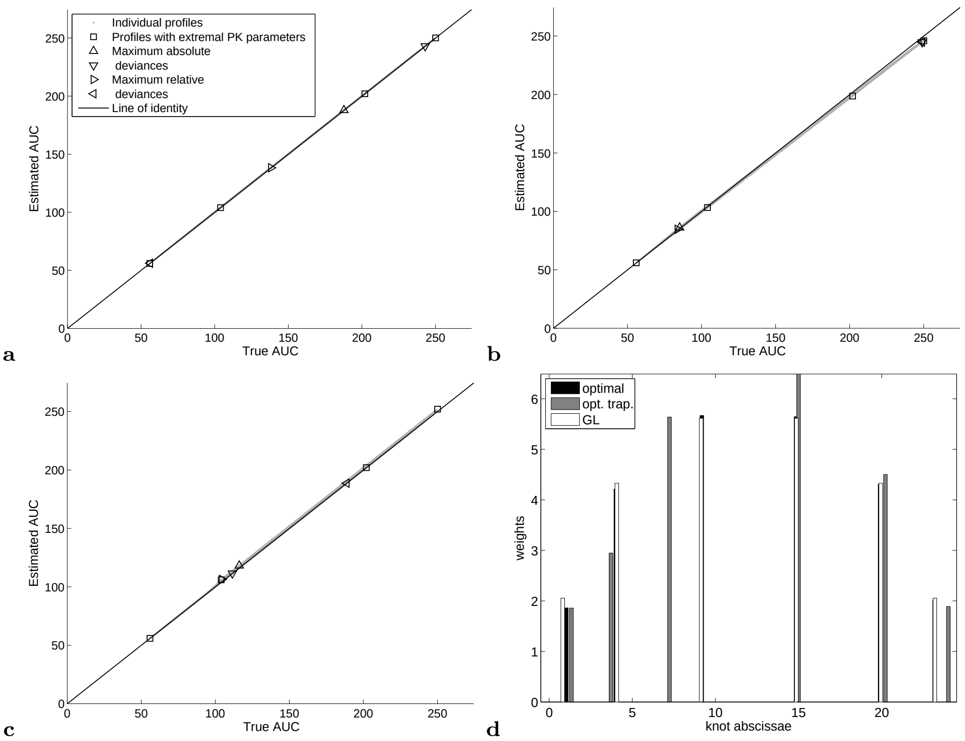

Fig. 3 Performance of the investigated methods for Model 1 with the relative risk,n=6 and c_v=0 (no random error). **a-c**: true vs estimated AUC by optimal, optimal trapezoid and GL or CC method, respectively; **d**: knots and weights of these methods.

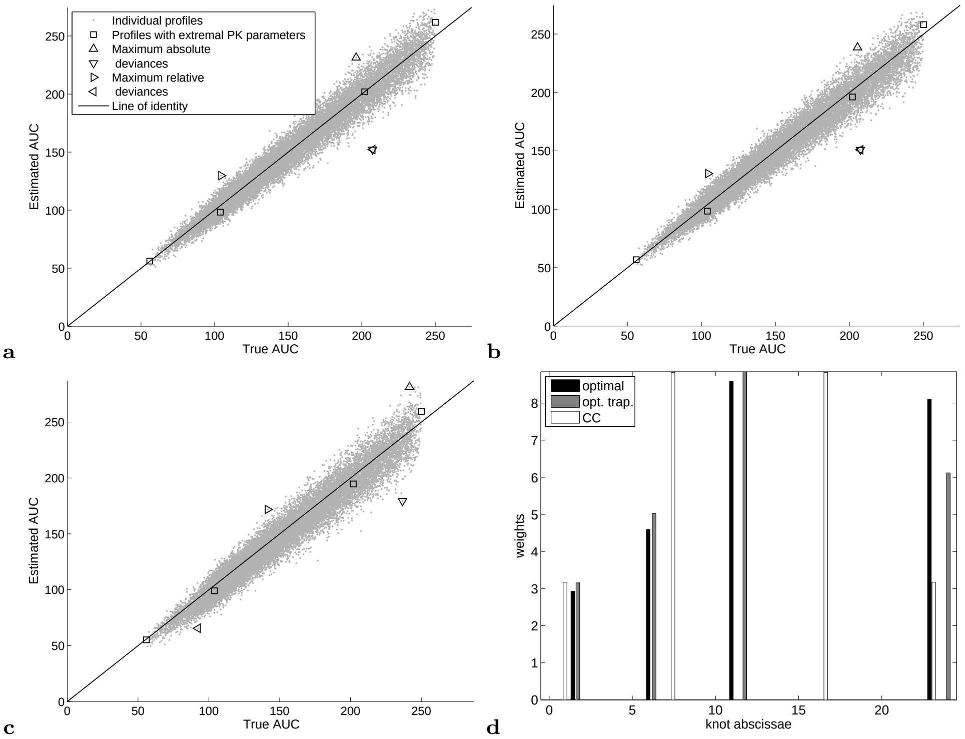

Fig. 4 Performance of the investigated methods for Model 1 with the relative risk,n=4 and c_v=0.10. **a-c**: true vs estimated AUC by optimal, optimal trapezoid and GL or CC method, respectively; **d**: knots and weights of these methods.

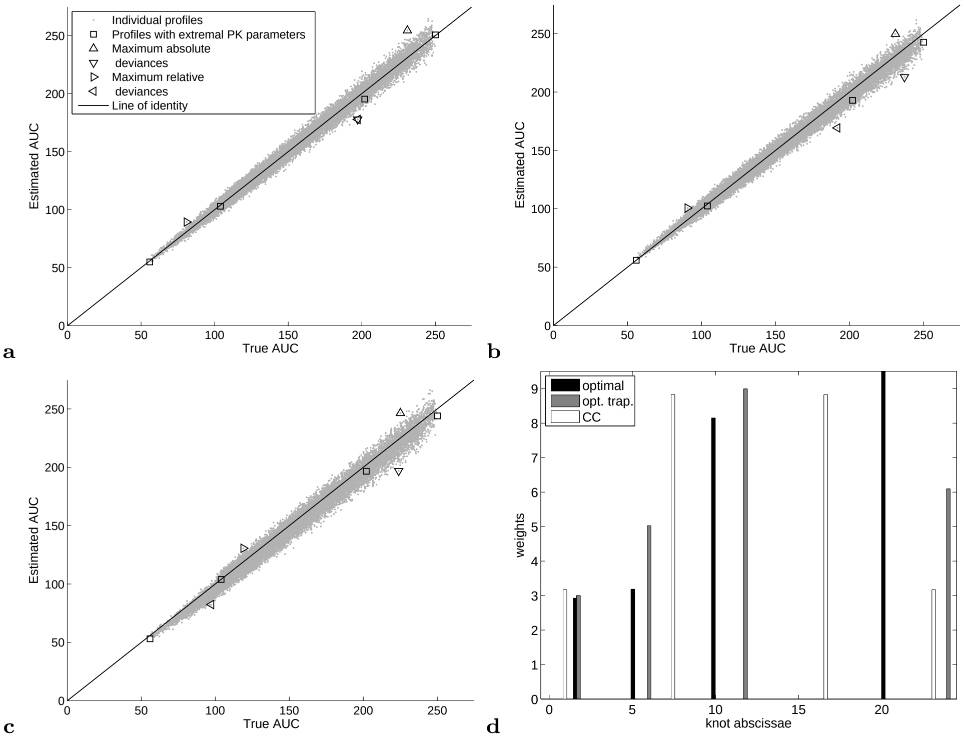

Fig. 5 Performance of the investigated methods for Model 1 with the relative risk,n=4 and c_v=0.05. **a-c**: true vs estimated AUC by optimal, optimal trapezoid and GL or CC method, respectively; **d**: knots and weights of these methods.

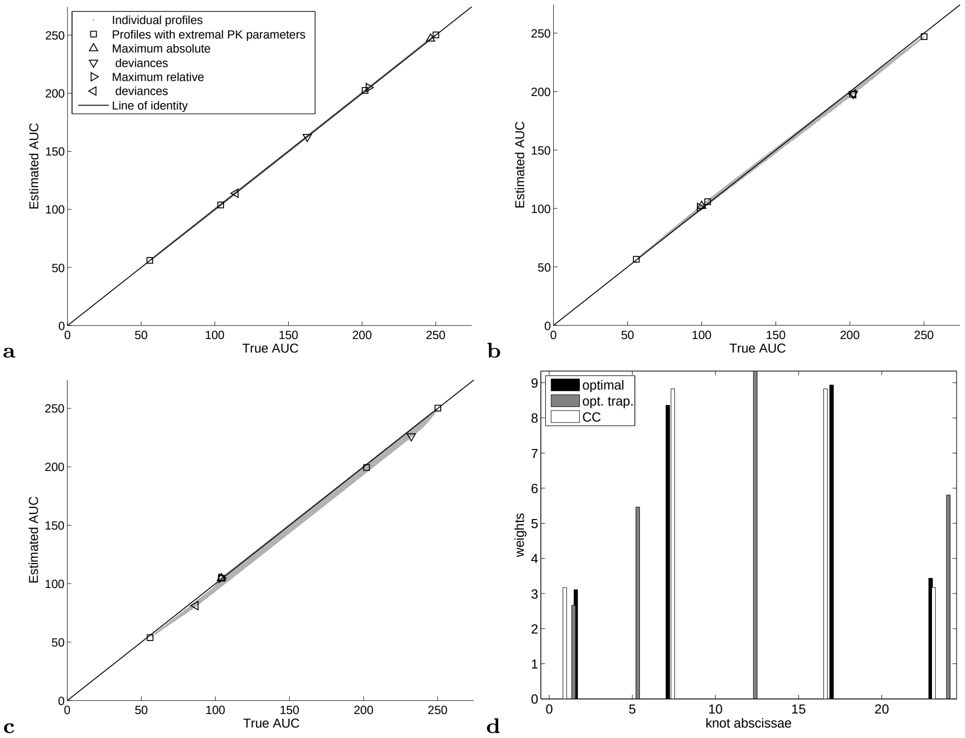

Fig. 6 Performance of the investigated methods for Model 1 with the relative risk,n=4 and c_v=0 (no random error). **a-c**: true vs estimated AUC by optimal, optimal trapezoid and GL or CC method, respectively; **d**: knots and weights of these methods.

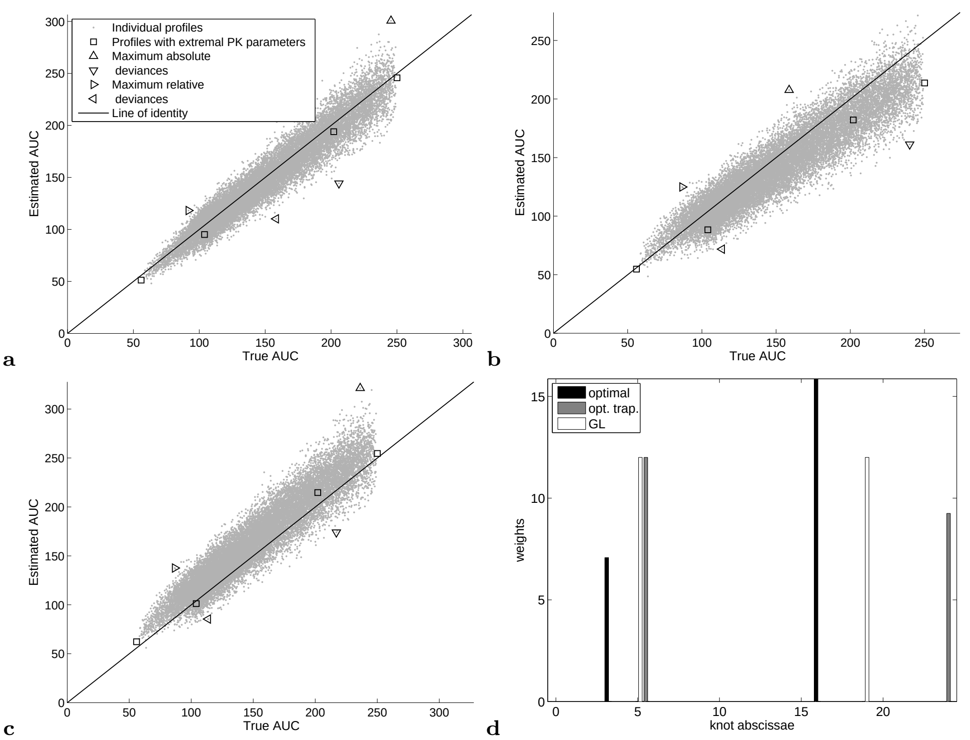

Fig. 7 Performance of the investigated methods for Model 1 with the relative risk,n=2 and c_v=0.10. **a-c**: true vs estimated AUC by optimal, optimal trapezoid and GL or CC method, respectively; **d**: knots and weights of these methods.

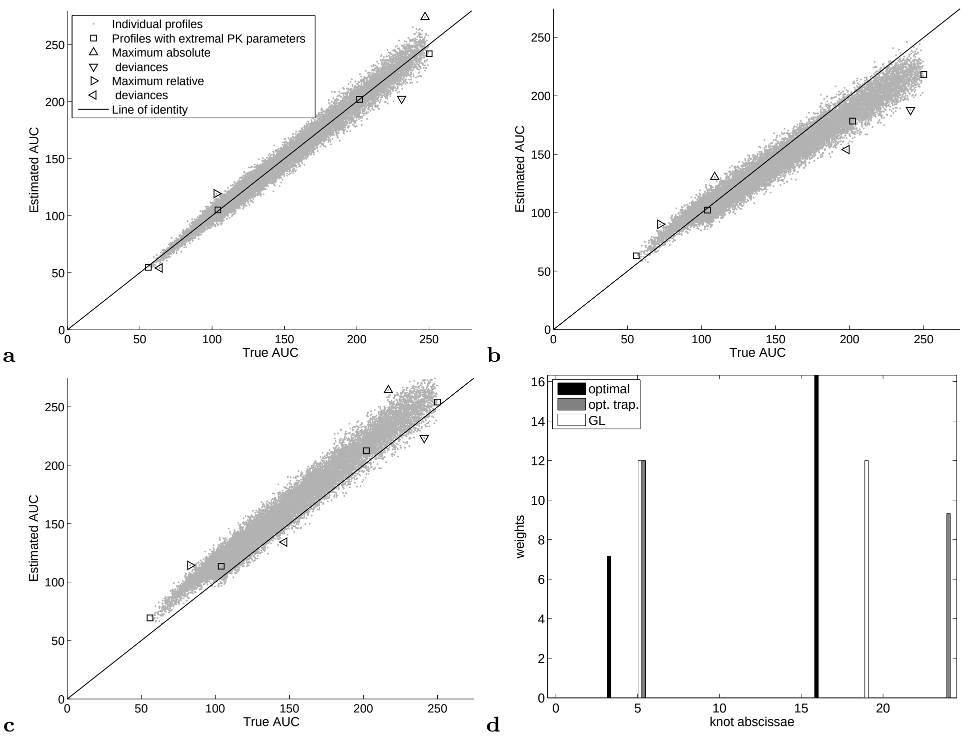

Fig. 8 Performance of the investigated methods for Model 1 with the relative risk,n=2 and c_v=0.05. **a-c**: true vs estimated AUC by optimal, optimal trapezoid and GL or CC method, respectively; **d**: knots and weights of these methods.

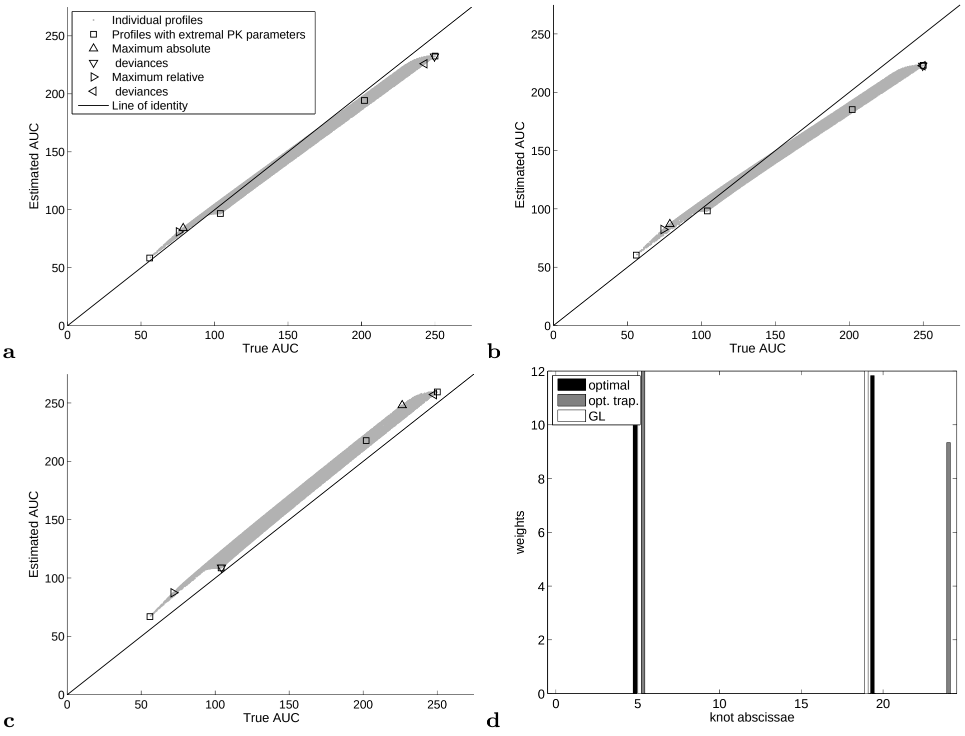

Fig. 9 Performance of the investigated methods for Model 1 with the relative risk,n=2 and c_v=0 (no random error). **a-c**: true vs estimated AUC by optimal, optimal trapezoid and GL or CC method, respectively; **d**: knots and weights of these methods.

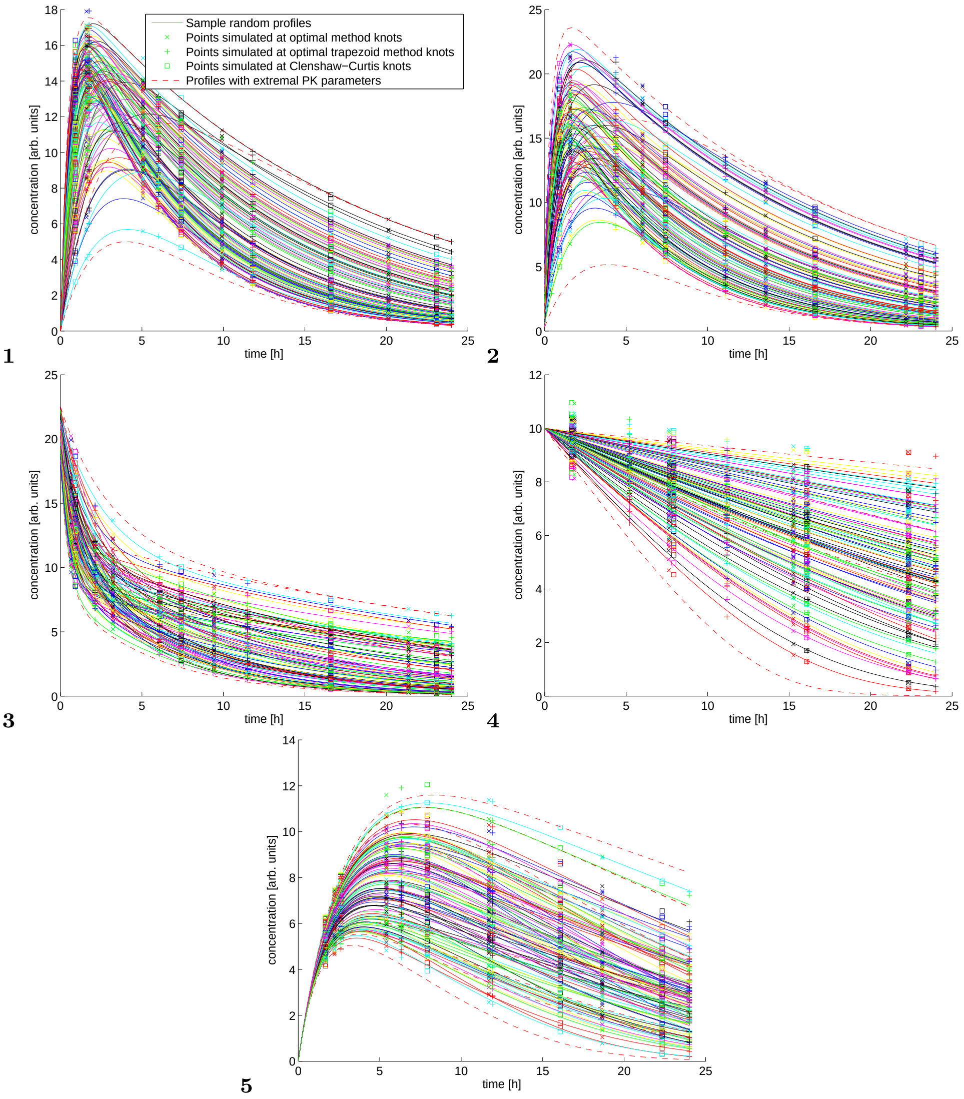

Fig. 10 Spaghetti plots for $n = 4$ and c_v =5% across models **1**–**5** with relative risk
